# Preliminary validation of a Urinary Symptom Questionnaire for individuals with Neuropathic Bladder using Intermittent Catheterization (USQNB-IC): A patient-centered patient reported outcome

**DOI:** 10.1371/journal.pone.0197568

**Published:** 2018-07-10

**Authors:** Rochelle E. Tractenberg, Suzanne L. Groah, Amanda K. Rounds, Inger H. Ljungberg, Manon M. Schladen

**Affiliations:** 1 Collaborative for Research on Outcomes and –Metrics; and Department of Neurology; Biostatistics, Bioinformatics & Biomathematics, Georgetown University Medical Center, Washington, DC, United States of America; 2 Department of Rehabilitation Medicine, Georgetown University Medical Center, Washington, DC, United States of America; 3 MedStar National Rehabilitation Hospital, Washington, DC, United States of America; 4 MedStar Health Research Health Institute, Hyattsville, Maryland, United States of America; Northwestern University, UNITED STATES

## Abstract

**Background:**

We developed a Urinary Symptom Questionnaire for individuals with neurogenic bladder due to spinal cord injury (SCI) and spina bifida (SB) who manage their bladders with intermittent catheterization, the USQNB-IC. This project followed an approach to patient-centered patient reported outcomes development that we created and published in 2017, specifically to ensure the primacy of the patient’s perspective and experience.

**Participants:**

Two sets of responses were collected from individuals with neurogenic bladder due to either SCI (n = 336) and SB (patients, n = 179; and caregivers of patients with NB, n = 66), and three sets of “controls”, individuals with neurogenic bladder who do *not* have a history of UTIs (n = 49) individuals with chronic mobility impairments (*neither* SCI nor SB) and without neurogenic bladder (n = 46), and those with no mobility impairment, no neurogenic bladder, and no history of UTIs (n = 64).

**Method:**

Data were collected from all respondents to estimate these psychometric or measurement domains characterizing a health related PRO: Reliability (minimization of measurement error; internal consistency or interrelatedness of the items; and maximization of variability that is due to “true” difference between levels of the symptoms across patients), and validity (*content*, reflection of the construct to be measured; *face*, recognizability of the contents as representing the construct to be measured; *structural*, the extent to which the instrument captures recognizable dimensions of the construct to be measured; and *criterion*, association with a gold standard).

**Results:**

Evidence from these five groups of respondents suggest the instrument has face, content, criterion, convergent, and divergent validity, as well as reliability. The items were all more descriptive of our patient (focus) groups and were only weakly endorsed by the control groups.

**Conclusions:**

The instrument is unique in its emphasis on, and origination from, the lived experiences of patients with neurogenic bladder who use intermittent catheterization; this preliminary psychometric evidence suggests the instrument could be useful for research and in the clinic. These results justify further development of the instrument, including formal exploration of the scoring and estimation of responsivity of these items to clinical interventions as well as patient-directed self care.

## Introduction

Urinary tract infection (UTI) is a major chronic and recurrent health condition for people with neurogenic bladder, as well as being a major worldwide public health problem.[1–4] Neurogenic bladder is also associated with a disproportionately high risk of genitourinary complications, including bladder and kidney infections,[1,5–7] calculi,[8,9] and bladder cancer,[5,10] among others. While neurogenic bladder can result from any trauma or disease of the brain or spinal cord, people with spinal cord injury (SCI) and spina bifida (SB) are nearly universally affected. Urinary tract infections (UTIs) and renal damage were historically the most common cause of death for people with SCI and SB[7], and while that early mortality has declined with improved genitourinary management, UTIs remain the most common cause of emergency department visits[8] and rehospitalization[11] among people with neurogenic bladder.[5,12–14]

*Identification* of a UTI is paramount in prevention and management efforts, and that is complicated in the patient with neurogenic bladder by several factors. One of the most important challenges in early detection of a UTI is that the patient needs to identify symptoms that may be indicative of UTI, and then those symptoms need to be understood by a health care professional to determine whether the symptoms represent an actual UTI, non-UTI urinary symptoms, or symptoms due to another cause. What constitutes symptoms indicative of UTI, which are often significantly altered in people who have sensory impairment relative to groups from whom neurologic function is intact, is not clearly defined for people with neurogenic bladder. These gaps are long-standing and have resulted in attempts to address diagnostic shortcomings by the National Institute of Disability and Rehabilitation Research (1992), the Agency for Healthcare Research and Quality (1999), and the Infectious Diseases Society of America (2009). Without exception, all of these guidelines note the lack of high quality supporting evidence for their use, and each calls for research addressing these challenges in people with neurogenic bladder.

To that end, our research team is developing a series of Urinary Symptom Questionnaires for people with neurogenic bladder according to bladder management method. This report specifically refers to the development of a Urinary Symptom Questionnaire (USQ) for individuals with neurogenic bladder (NB) due to SCI and SB, and who manage their bladders with *intermittent catheterization* (IC) (USQNB-IC), focusing on upper and lower urinary tract signs and symptoms. While historically, UTI diagnostic criteria and instrument development have been driven primarily by clinical perspectives[15], without accommodating the patient’s lived experience, this instrument was developed following an approach that we developed[16] specifically to ensure the primacy of the patient’s perspective and experience with neurogenic bladder. Our objective has been to create an instrument that can promote patients’ self-management of urinary signs and symptoms, and which may be useful for preventative, comparative effectiveness, and diagnostic research around urinary signs and symptoms, or possibly of UTIs, in the future. As such, the patient’s report is essential; however, rather than focusing on quality of life, as many “patient reported” perspectives on UTI have done[17–19], our overall approach has had a purposeful focus on signs and symptoms in the patients’ experience that could potentially bear on interventions and their development and validation in the future.

Based on clinical experience, we suspect a urinary symptom instrument will differ depending on the degree of neurogenic impairment of the bladder, and because bladder management method (indwelling catheterization, intermittent catheterization, and voiding, in decreasing order of severity) is, to some extent, a proxy for degree of neurogenic bladder dysfunction. This initial effort is focused on people with neurogenic bladder who manage their bladders with intermittent catheters, and here we report on the preliminary validation evidence for the USQNB-IC for this population. This article follows and utilizes the recommendations and definitions of the international Consensus-based Standards for the selection of health Measurement Instruments, COSMIN[20] to document the measurement properties of the new USQNB-IC.

## Methods

**Fig 1:**
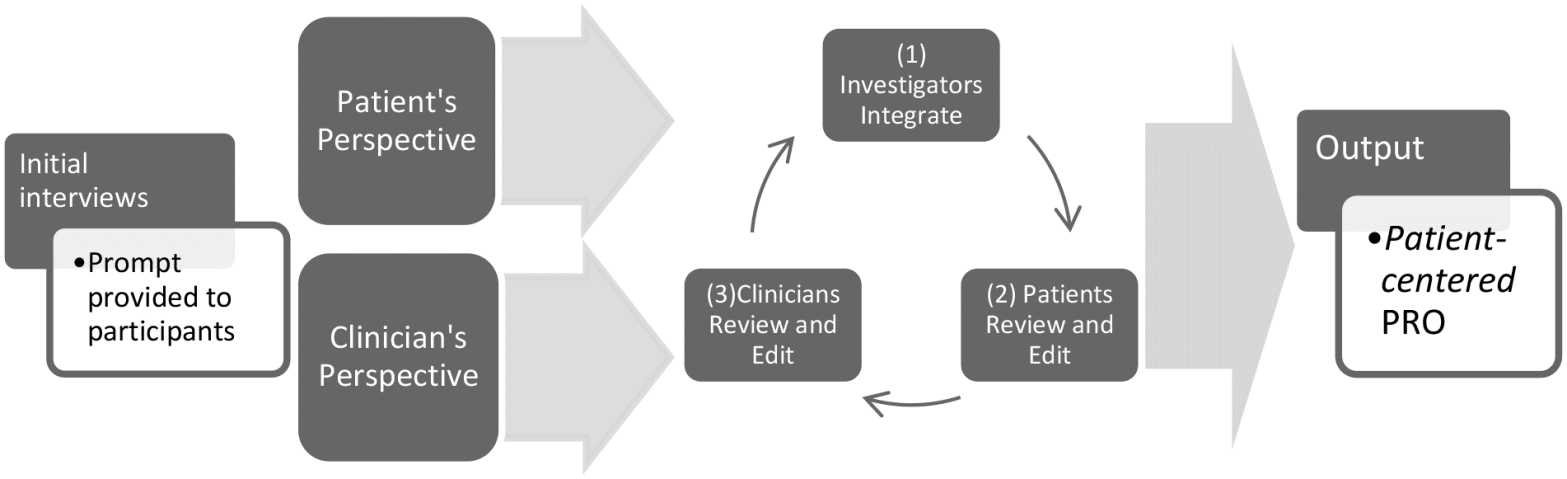
Framework for developing a patient-*centered*, patient reported outcome. (adapted from Tractenberg, et al. (2017)[16], Fig 2 –with permission).

Approval for all parts of this study was received from the MedStar National Rehabilitation Hospital Institutional Review Board (IRB # 2013–187). Participants were recruited by advertising the need for responses on this new instrument through Facebook, and via email and otherwise with the assistance of the national (U.S.) advocacy networks in spinal cord injury and spina bifida. All of these outreach and recruitment efforts were advertisements seeking respondents with NB who use intermittent catheters to visit the URL we established for data collection. Within 10 months, nearly 600 responses had been obtained from individuals with spinal cord injury, spina bifida, and their caregivers. We then sought responses from participants in our validation (divergent/convergent) populations, described below. No personal or identifying data were collected from any respondent, and initiating responses on the survey was deemed sufficient consent by the IRB. We developed the USQNB-IC following a new model for the creation of patient-centered patient reported outcomes[16] shown in Fig 1.

### Methods: Materials

An instrument with 29 items resulted from a total of 13 focus groups with eligible patients (with neurogenic bladder due to SCI or SB, and who use intermittent catheters) or their caregivers, from around the country, plus iterative focused discussions with a core of four clinicians with expertise in this domain (as described in Tractenberg et al. 2017).[16] As described below, a national sample of people with neurogenic bladder who manage their bladders with intermittent catheterization were recruited from patient populations of SCI (N = 336) and SB (N = 178), plus caregivers of people with SB (N = 66). Participants were recruited to complete the instrument online, using SurveyMonkey. Each item was presented as a query about whether the respondent had experienced it during the past year (yes/no), with three additional required responses: average frequency (0–365); average severity (usually not at all severe; usually somewhat severe; usually severe; always very severe); and average impact on, or importance in, daily life (rarely affects my actions or decisions to go about my daily life; sometimes affects my actions or decisions to go about my daily life; usually affects my actions or decisions to go about my daily life; always affects my actions or decisions to go about my daily life). The analyses described below were focused on the endorsement (yes/no) of items as sources of validity evidence for the instrument and its makeup. Once preliminary validity evidence is established, we plan to revisit the frequency, severity, and impact ratings to develop and refine a scoring algorithm in a future article.

### Methods: Validation

Our analyses were planned to describe the validity of the USQNB-IC using classical psychometric methods.[21] The COSMIN report identifies four key psychometric or measurement domains on which a health related PRO should be characterized: Reliability (minimization of measurement error; internal consistency or interrelatedness of the items; and maximization of variability that is due to “true” difference between levels of the symptoms across patients), validity (*content*, reflection of the construct to be measured; *face*, recognizability of the contents as representing the construct to be measured; *structural*, the extent to which the instrument captures recognizable dimensions of the construct to be measured; and *criterion*, association with a gold standard), cross-cultural validity, and the interpretability of scoring. Cross-cultural validity would not be relevant if any of the other measurement properties are not met, so we did not address this type of validity in this study. Similarly, responsiveness, defined by the COSMIN criteria as the sensitivity of an instrument to change in the construct of interest, was also not evaluated in this study because like cross-cultural validity, that would be premature.

The development of this instrument was characterized by a focus on face and content validity from both the clinician and the patient perspectives (Fig 1, above), thus this report augments that content and face validity evidence. Because there is, by their origins, no diagnostic “gold standard” for what these signs and symptoms represent, our evidence on convergent and divergent validity was classified as supporting both “reliability” and “criterion validity”, as described in the next section.

Importantly, the COSMIN requirements for “structural validity”, which they defined as dimensionality, are not consistent with either the approach to patient-centered patient reported outcomes that we followed, nor with the early stage of our instrument development. Specifically, for formal structural dimensionality to be a feature of a PRO at this early stage, it would need to have been developed following procedures outlined by Bollen (1989), with a measurement model in mind.[15,21] Instead, our instrument was developed (as noted) with *no* measurement model in mind, but instead with the specific intention of capturing the patient experience with high fidelity. Thus, the dimensionality on which we can report relates to the aspects of urinary signs and symptoms, and not to specific causal or response-generating features of the items on the instrument. We estimated internal consistency of the items with Cronbach’s alpha, which is a function of the correlation of each item with the total score that takes the total score variability into account, and provides an estimate of the shared covariance of items on the instrument–but does not relate to the instrument’s dimensionality or response-generating mechanism.[22,23]

In this report we describe new evidence of face and content validity as well as estimating internal consistency (COSMIN defined “reliability”) with Cronbach’s alpha, dimensionality (COSMIN defined “construct validity”) with exploratory common factor analysis, modeling the covariance of items; and inferred causal model with Bayesian networks, modeling the shared Shannon Information. Given the early stage of this instrument, we demonstrate both construct and criterion validity (which COSMIN defined as including divergent and convergent validity) with distributions of scores (total and item endorsements) for the target population of individuals with neurogenic bladder who manage their bladders with intermittent catheterization, and also with data from individuals with neurogenic bladder who do *not* have a history of UTIs, individuals with chronic mobility impairments (*neither* SCI nor SB) and without neurogenic bladder, and those with no mobility impairment, no neurogenic bladder, and no history of UTIs.

Table 1 below recapitulates the COSMIN measurement properties and describes the methods by which these were analyzed in this article.

**Table 1:** COSMIN^20^ criteria and how they were assessed in this study.

COSMIN construct	Definition^20^	Approach/analysis	Comment
Reliability-internal consistency	Degree of interrelatedness among items	Principal axis factoring (PAF) on tetrachoric correlations (between 0/1 endorsements); Cronbach’s alpha; inferred Bayesian Network	PAF extracts the commonalities; Cronbach’s alpha summarizes the shared covariance with respect to total variance.
Validity- content	Degree to which instrument measures the construct it targets	By development & design (specifically created to achieve this purpose)	Previously published^16^; detailed descriptive statistics
Validity–face	Degree to which items “look” as if they are an adequate reflection of the target construct	By development & design, iteratively eliciting and obtaining input from patients and clinicians.	Previously published^16^; detailed descriptive statistics
Validity- construct	Degree to which the scores are consistent with expected similarities (convergent) and differences (divergent) between groups	Divergent and convergent validation samples; comparisons of endorsement rates; and total numbers of items endorsed across groups.	Convergent validity: similar endorsement rates across NB groups; Divergent validity: endorsement rates for people without NB similar to each other, dissimilar to NB groups.
Validity-criterion	Degree to which the scores reflect a “gold standard”	See construct validity; also, by attribution of each item by respondents to “having a UTI”.	No diagnostic gold standard; we use convergent and divergent validity data instead.
Validity-structural	Degree to which the scores are an adequate reflection of the dimensionality of the target construct	Bayesian Network (BN) to uncover associated signs and symptoms; Principal axis factoring (PAF) on tetrachoric correlations (between 0/1 endorsements for full group)	BN is not a causal model; PAF is explicitly causal, but not entirely aligned with our patient- and not measurement model- centered approach.
Interpretability	Degree to which a qualitative meaning (patient/clinician perspectives) to the scores	Alignment of the endorsement rates with clinical practice guidelines	Detailed descriptive statistics

### Methods: Target patient population participants and recruitment

A nationally representative sample of individuals with neurogenic bladder due to either SCI or SB was recruited to complete the survey. We also obtained responses from individuals caring for people with SCI or SB. These samples represent the target populations as well as convergent validity data, and are described below.

*Inclusion Criteria*: Inclusion criteria for participants in the national sample with SCI: 1) age≥18 years; 2) SCI at least 1-year duration; 3) neurogenic bladder, as reported by respondents; 4) utilizing intermittent catheterization for bladder management; 5) and a history of 2 or more UTIs in the past year. Inclusion criteria for participants in the national sample with SB were: 1) age≥ 18 years; 2) neurogenic bladder, as reported by respondents; 3) utilization of intermittent catheterization for bladder management; 4) and a history of 2 or more UTIs in the past year. The inclusion criterion for participants in the national sample who were caring for a dependent with SB was only that they were a caregiver for an individual with SB or SCI.

Our consumer partner, United Spinal Association, assisted in recruiting nationally-representative samples of adults with SCI (N = 336), adults with SB (SB; N = 179) caregivers of children with SB (CG; N = 66).

### Methods: Divergent validity participant recruitment

Individuals with neurogenic bladder who did *not* self-report a history of UTIs (however the respondent defined this), individuals with chronic mobility impairments (*neither* SCI nor SB) and *without* neurogenic bladder, and individuals with no mobility impairment, no neurogenic bladder, and no history of UTIs were recruited largely from an inner city rehabilitation hospital to complete the same instrument.

### Methods: Data analysis

Statistical analyses were carried out using SPSS v. 24[24] for descriptive statistics and to estimate the variance explained by the exploratory factor analysis; exploratory factor analysis with tetrachoric correlations was carried out in MPlus v. 8 (Statsoft, Los Angeles, CA). BayesiaLab v. 6 (Bayesia S.A.S., Laval, France) was used for the Bayesian network modeling: unsupervised structural learning (searching for the overall data structure) using a Maximum Weight Spanning Tree (MWST) learning algorithm to constrain the structure so that only one “parent” per variable is identified. Minimum Description Length (MDL–information, not probability) was used for scoring because linearity in relationships cannot be safely assumed for these items. The resulting tree was visualized in automatic layout for interpretability (features discussed in Ch. 7 of Conrady & Jouffe, 2015).[25] The data analyzed in this manuscript are available as Supporting information files.

## Results

The results are presented in two parts. First, descriptive results for each of our three responding diagnostic groups are presented, followed by the descriptive data from our three “control” groups. Then, we describe the validity and reliability results for the instrument based on these respondents.

### Results: Descriptive statistics, SCI

Complete and valid (i.e., from eligible respondents) responses to the USQNB-IC were obtained from 336 individuals with spinal cord injury (SCI). On average, respondents with neurogenic bladder were 46.7 years old (sd: 13.64y; range 18–75) and had been living with their injury (and neurogenic bladder) for an average of 14.7 years (sd: 12.5; range 1–62 years). Of this sample, 45.5% were women and 30.1% reported that they reuse catheters; we did not obtain the level of injury but participants did report whether their injury was complete (39.9%), incomplete (53.9%), or unknown (6.8%).

### Results: Descriptive statistics, SB

Complete and valid responses to the USQNB-IC were obtained from 179 individuals with spina bifida (SB). On average, respondents were 35.3 years old (sd: 11.7 y; range 18–65). Of this sample, 72% were women and 42.8% reported that they reuse catheters.

### Results: Descriptive statistics, caregivers of patients

The most difficult group to recruit were caregivers of individuals with SB or SCI. Complete and valid responses to the USQNB-IC were obtained from 66 individuals who are currently caring for an individual with SB or SCI. On average, the individual being cared for was 12.4 years old (sd: 9.4 y; range 1–44). Of those being cared for in this sample, 55% were female and 6% reported that they reuse catheters.

### Results: Descriptive statistics, three control groups

The total number of control respondents was 160, with 53.75% female overall (36% females with NB, no UTI; 43% females with mobility impairment, without NB, no UTI; and 75% female no mobility impairment, without NB, no UTI). The average age was 55.7 years (SD = 16.8) years (49.5 years (SD = 15.3) for 47 participants with NB, no UTI; 60.3 years (SD = 15.7) for 49 participants with mobility impairment, without NB, no UTI; and 56.6 years (SD = 17.4) for 64 participants with no mobility impairment, without NB, no UTI). Of these 160 individuals, 16 use catheters.

### Results: Validity evidence, reliability

The approach to validation was derived from Mokkink et al. (2010)[20], as discussed above; content and face validity are conferred by the process by which the instrument was created (Fig 1).[16] Table 2 summarizes the COSMIN criteria and our results following the methods outlined in Table 1.

**Table 2:** COSMIN^20^ reliability evidence: Patient groups.

COSMIN construct:	Grouping:			
Reliability-internal	Whole Group (N = 581)	SCI (N = 336)	SB (N = 179)	SB CG (N = 66)
Principal Axis Factoring/oblimin*	PAF: 8 correlated factors explain 35.6% of the variance §			
Cronbach’s alpha	.846	.843	.844	.865
ICC	.846 (95% CI .827-.864)	ICC = .846 (95% CI .817-.866)	ICC = .845 (95% CI .810-.876)	ICC = .869 (95% CI .819-.910)

Notes:

* Oblimin (correlated factors) rotation was planned; factors were significantly correlated (data not shown).

^§^ Percent of variance explained is *estimated* from PAF carried out on non-tetrachoric correlation matrix in SPSS (v. 25) because variances are required to compute the explained variability, but no formula exists to estimate this for tetrachoric correlations.

### Results: Exploratory factor model and inferred causal network

Principal axis factoring (PAF) with oblimin rotation extracted eight factors from the tetrachoric correlation matrix of endorsement rates of the overall sample (N = 581; data not shown). This model fit the data well (root mean square of the approximation, RMSEA = 0.024, 95% CI: 0.016–0.031; confirmatory factor index, CFI = 0.985; Tucker’s factor index, TFI = 0.971; standardized root mean square of the residuals 0.037). The 8-factor model fit significantly better than the 7 (chi square 52.8 with 22 degrees of freedom, p = 0.002) and all larger models up to 8 fit significantly better than models with fewer factors (data not shown but all p<0.001); however, we estimated that just 35.6% of the total variance in this system was explained by the 8- factor model using the PAF method with oblimin rotation based on the Pearson Correlation matrix in SPSS. Oblimin was used because of the high likelihood of correlated factors (given the origins of the items within focus group sessions guided by a single common script); many of the eight extracted factors were correlated with p<0.05; these data are not shown because although the 8 factor model fit well according to the fit indices, a) an 8 factor model did not have much interpretability; b) the estimated variance explained was inadequate (35.6%)–yet this was greater than for any smaller model; and c) we did not hypothesize a meaningful measurement model given the time frame of our questions and the origins of these items (i.e., without a measurement model in mind).

The sub-samples were too small (apart from SCI) to re-run this extraction procedure with interpretable results; however it was not hypothesized that collapsing over diagnostic group was the cause of the limited explanatory power of the modeling. Given the origins of these items (i.e., not following Bollen 1989/no measurement model in mind) and the fact that all queries were formulated for “the past year”, the lack of a coherent causal measurement model that explaining a majority of the variability among the three neurogenic bladder response groups was not surprising.

We next used inferred Bayesian networks (Bayesialab v. 6, Bayesia S.A.S., Laval, France) to discover evidence of underlying structure for these items. This method is not parametric, does not use or require inferences, and uses information instead of probability for estimating associations. The results are, therefore, associative and not specifically causal. This method allowed us to identify possible clusterings of symptoms, without sample sizes or parameter estimation precision being the issue that they can be in an inference-, or estimation-, oriented method like PAF. Like the PAF analysis above, the Bayesian network was inferred based on the yes/no endorsement of all items for the full sample using unsupervised learning and the maximum spanning tree option. This method was used to uncover basic structure, but limit the associations that appeared to a single-neighbor (i.e., a Markov Blanket approach). This method is similar to the employment of rotation in factor analysis solutions for “simple structure”.

**Fig 2:**
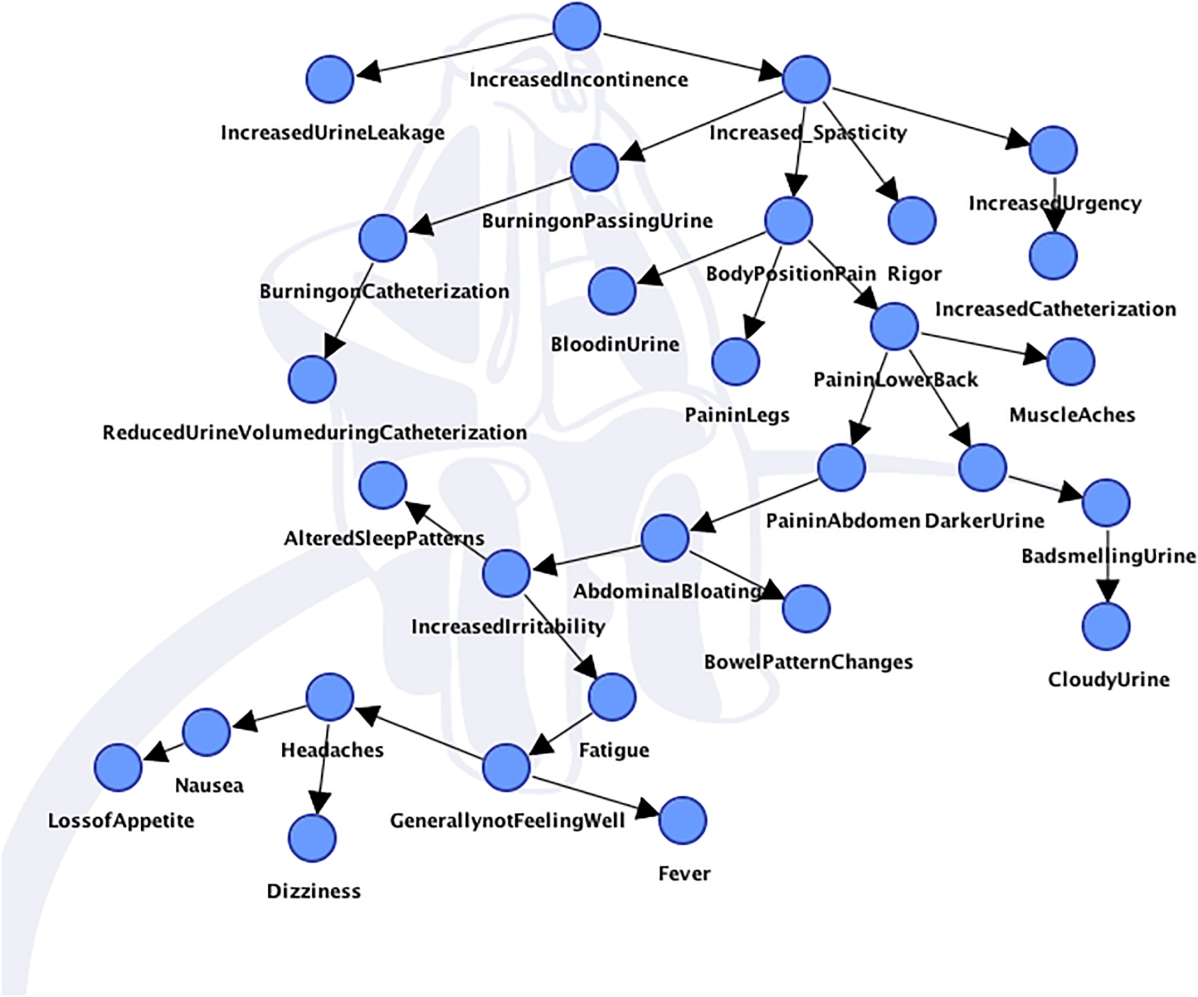
Bayesian network of USQNB-IC items, full sample.

We discovered a network with a complex structure from the full sample of 581. The network is represented in Fig 2 below.

The network shown in Fig 2 highlights the complexity of the lived experience of urinary signs and symptoms in neurogenic bladder. Importantly, as anticipated (see comments above), the structure does not show a single causal or originating factor for all or even some of the symptoms; this has both treatment (clinician) and management (patient) implications, as well as highlighting critical characteristics of the patient’s experience for clinical research and clinical trials of future interventions for UTI. For example, “incontinence” and “frequency of spasms” are directly or indirectly related to the majority of symptoms; therefore, if interventions address these two symptoms, they may have the most distributed effects. By contrast, the associations of “generally not feeling well” are more limited (i.e., only associated with fatigue, fever, headaches, etc.), and therefore, an intervention that addressed “generally not feeling well” might have much more limited effects than one addressing incontinence. At this preliminary stage of validation we asked whether each item had been experienced within one year. Our follow-up research questions will include whether each item was experienced within any one-week period, because that is the putative timeframe for use of the instrument in research or treatment. Because of the confounding effects of the timeframe of the questions, and because the measurement model structure (e.g., path weights, factor makeup) was not our purpose with this model, we did not estimate fit, factors, or strengths of associations between any of the items.

That this network was readily interpretable is additional evidence that the USQ-NB-IC has sufficient structural validity for continuing the evaluation of its measurement properties. The interpretability of this network also contributes to the reliability evidence for the instrument.

The instrument demonstrates reliability and construct validity as discussed in the preceding section. Content and face validity are conferred by the process by which the instrument was created (Fig 1). Additional content and face validity evidence arises from an examination of endorsement rates across the three groups of nationally-representative patients with neurogenic bladder in our sample. Table 3 provides the presenting the endorsement rates (number of respondents identifying having experienced the symptom within the past year/total number of respondents in that group) by persons with neurogenic bladder for each symptom on the USQNB-IC in the preceding 12 months.

**Table 3:** Item endorsement (in percent) by group.

ITEM:	SCI % endorsement (n = 336)	SB % endorsement (n = 179)	CG % endorsement (n = 66)
Cloudy Urine	81%	64%	82%
Bad smelling Urine	81	71	79
Darker Urine	73	64	68
Blood in Urine	32	22	21
Increased frequency of Spasms	58	50	35
Increased urgency	53	58	30
Increased Catheterization	49	46	33
Increased Incontinence	63	56	50
Increased Urine Leakage	50	60	53
Reduced Urine Volume during Catheterization	38	24	41
Pain in Abdomen	26	22	29
Pain in Lower Back	32	32	29
Pain in Legs	29	30	18
Burning on Catheterization	19	23	21
Burning on Passing Urine	13	23	11
Body Position Pain	37	31	15
Fever	45	43	53
Fatigue	61	74	44
Generally not Feeling Well	66	60	55
Muscle Aches	54	51	32
Altered Sleep patterns	48	39	29
Increased Irritability	35	42	39
Headaches	44	59	47
Dizziness	27	31	18
Rigor	44	11	12
Abdominal Bloating	38	38	24
Nausea	33	35	44
Loss of Appetite	29	26	33
Bowel Pattern Changes	43	44	36
Mean, SD total # endorsed (range)	13.0, SD = 5.8 (range 1–29)	12.3, SD = 5.9 (range 0–29)	10.8, SD = 6.1 (range 1–25)

Endorsement rates are similar for individuals with SCI & SB, whereas endorsement by item was slightly different for the caregivers. The endorsement rates for each item on the instrument were at least 10% in every group, supporting a claim of face validity across these participants since all items are perceptible to these three patient groups.

Table 3 shows that the endorsement rates for the items are generally similar across groups, except for rigor (far more frequently endorsed by SCI). We did not test for significant differences across groups due to a) a need for correction for 29 comparisons (reducing the likelihood that any would remain significant) and b) our assumption that the focus groups and focus group facilitators, who were consumers themselves (as described previously[16]), were able to capture the lived experience of neurogenic bladder across etiology.

Four of the five most highly endorsed items were the same in each group: Darker urine; cloudy urine; bad smelling urine; and generally not feeling well. Fatigue was reported in the SB group with greater frequency than the other two groups. The fifth most frequently endorsed item was increased incontinence for SCI; increased leakage for SB and fever for CG.

The similarities of endorsement rates across groups offers content and face validity evidence, together with convergent validity and some reliability evidence. It also suggests that neurogenic bladder—which was the defining feature of the focus groups from which the symptoms were derived–*does* have a set of “signs and symptoms” that neurogenic bladder patients may experience. The focus groups convened neurogenic bladder patients from diverse etiologies and their input created a set of signs and symptoms that the national sample of neurogenic bladder respondents recognized. The similarities suggest the instrument is relevant/resonant for neurogenic bladder respondents who manage their bladders with intermittent catheters, also contributing evidence of face and content validity.

Table 4 presents the endorsement rates across our control groups. These groups were recruited (as noted above) to evaluate the prevalence of these symptoms for other groups (not SCI or SB) with mobility impairments without neuropathic bladder, those with neuropathic bladder but who do not have frequent UTIs, and finally from those without mobility impairment, neuropathic bladder, or frequent UTIs. Importantly, in Table 4, endorsement of items was not simply interpretable as “yes/no” because for some of these participants the items had no possibility of endorsement. That is, for divergent validity participants, catheterization was very rare (just 16 of 160) whereas all participants in the USQNB-IC groups did use intermittent catheterization. Thus, the endorsement rates in Table 4 are given together with the number of respondents in any given group for whom the item was possible to endorse.

**Table 4:** Item endorsement in percentage, by group, with the number of respondents endorsing each item in parentheses.

ITEM:	Mobility Impairment, No NB (n = 49)	NB, no UTIs (n = 47)	No mobility impairment, no NB, no UTIs (n = 64)	ALL controls (n = 160)
Cloudy Urine	4% (n = 49)	15% (n = 47)	14% (n = 64)	11% (n = 160)
Bad smelling Urine	16% (49)	15% (46)	25% (64)	19% (159)
Darker Urine	33% (49)	28% (47)	16% (64)	24% (160)
Blood in Urine	0% (49)	4% (46)	3% (64)	3% (159)
Increased Frequency of spasms	2% (49)	17% (46)	13% (64)	11% (159)
Increased urgency	41% (49)	39% (46)	49% (63)	44% (158)
Increased Catheterization	0% (1)	29% (14)	NA	27% (15)
Increased Incontinence	20% (49)	37% (46)	27% (62)	28% (157)
Increased Urine Leakage	12% (49)	27% (45)	45% (62)	29% (156)
Reduced Urine Volume during Catheterization	100% (1)	14% (14)	NA	20% (15)
Pain in Abdomen	8% (49)	11% (44)	17% (60)	12% (153)
Pain in Lower Back	20% (49)	23% (44)	52% (60)	33% (153)
Pain in Legs	41% (49)	30% (44)	32% (60)	34% (153)
Burning on Catheterization	0% (1)	21% (14)	NA	20% (15)
Burning on Passing Urine	0% (49)	2% (44)	8% (60)	4% (153)
Body Position Pain	12% (49)	23% (44)	15% (60)	16% (153)
Fever	10% (49)	7% (44)	22% (60)	14% (153)
Fatigue	39% (49)	44% (43)	38% (60)	40% (152)
Generally not Feeling Well	27% (49)	35% (43)	47% (60)	37% (152)
Muscle Aches	27% (49)	37% (43)	65% (60)	45% (152)
Altered Sleep patterns	16% (49)	28% (43)	42% (60)	30% (152)
Increased Irritability	18% (49)	23% (43)	45% (60)	30% (152)
Headaches	18% (49)	26% (43)	53% (60)	34% (152)
Dizziness	14% (49)	14% (43)	15% (60)	14% (152)
Rigor	22% (49)	35% (43)	5% (60)	19% (152)
Abdominal Bloating	8% (49)	16% (43)	31% (59)	19% (151)
Nausea	10% (49)	12% (43)	19% (59)	14% (151)
Loss of Appetite	18% (49)	9% (43)	12% (59)	13% (151)
Bowel Pattern Changes	8% (49)	16% (43)	31% (59)	19% (151)
Mean, SD total # endorsed (range)	4.6, SD = 4.3 (0–19)	5.62, SD = 5.1 (0–20)	7.0, SD = 4.4 (0–15)	5.8, SD = 4.7 (0–20)

In Table 4, the endorsement rates for “increased catheterization” and “burning on catheterization” are low, but the number of individuals responding to the item is also quite low. This is highlighted because these endorsement rates (%s) cannot be directly compared to the rates from Table 3, as the values in Table 4 for these items, at least, are based on dramatically smaller samples.

The last row in the table shows that the average number of endorsed items was 5.84 of 29 for the controls overall; this is less than half of the average for the patient groups (Table 3). The highest average number of endorsed items was in the group without mobility impairments and without neurogenic bladder (7.0, SD = 4.45). The lowest average number of endorsed items (4.55, SD = 4.27) was for those with mobility impairments who did not have neurogenic bladder.

There was agreement among the four most-frequently endorsed items among the control groups shown in Table 4. Increased urgency was among the top five most endorsed items in all three groups, while fatigue was in the top five for those with mobility impairment/no neurogenic bladder and those with neurogenic bladder but no UTIs; generally not feeling well and muscle aches were in the top five for both those with neurogenic bladder but no UTIs and those with neither UTIs, neurogenic bladder, nor mobility impairment. None of the endorsement rates for these items was as high as it was for any of the neurogenic bladder patient groups; the most striking difference between these control group endorsement rates and those from our national sample is for those with neurogenic bladder who have not experienced any (many) UTIs. Their rate of endorsement is the second lowest of the three control groups (with those who have neither mobility impairment nor neurogenic bladder having the highest average endorsement rate).

A final aspect of validity was explored by studying whether respondents tended to attribute the symptoms to a UTI. This feature of the instrument was challenging to conceptualize, not only because the frame of reference for endorsement was the previous year, but also because attribution, by the patient, of a symptom “to a UTI” could arise from a wide variety of ultimately unverifiable rationales. For example, a respondent could attribute a symptom to a UTI for any (one or more) of the following reasons:

1. Their clinician called it a UTI (whether or not this was confirmed);
2. Their clinician treated it with/prescribed antibiotics (whether or not this was consistent with clinical practice guidelines, which are noted to be based more on expert consensus than evidence)[26];
3. They self-diagnosed a UTI;
4. Previous experience with that or a similar symptom was clinically- or self- diagnosed as a UTI;
5. The symptom represented a deviation from normal and was attributed to UTI.

Because it is (and was) impossible to verify whether any experience was actually a UTI, we instead present results based on respondents’ assertions that the symptom was *never* associated with a UTI. This would therefore exclude all of the above possible associations with, or attributions to, a UTI. Therefore, Table 5 presents the proportions of respondents, collapsed over our national patients-with-neurogenic bladder samples (n = 581) and control (n = 160) groups. In each case, the proportions of those who endorsed each item and also *never* attributed it to a UTI are given.

**Table 5:** Proportions of respondents endorsing a USQ-NB item and *not* attributing it to a UTI.

ITEM:	Patients (SCI, SB, CG)	Controls
Cloudy Urine	13.3 *	83.3
Bad smelling Urine	13.0 *	93.1
Darker Urine	19.1 *	94.7
Blood in Urine	59.6	75.0
Increased Frequency of Spasms	32.0 *	87.5
Increased Urgency	35.9 *	97.0
Increased Catheterization	39.4 *	100
Increased Incontinence	27.9 *	100
Increased Urine Leakage	37.0 *	97.7
Reduced Urine Volume during Catheterization	52.7	100
Pain in Abdomen	64.8	84.2
Pain in Lower Back	57.1	100
Pain in Legs	60.1	100
Burning on Catheterization	71.0	100
Burning on Passing Urine	78.3	50
Body Position Pain	53.4	100
Fever	42.3 *	100
Fatigue	29.7 *	98.4
Generally not Feeling Well	24.7 *	94.6
Muscle Aches	32.3 *	98.5
Altered Sleep Patterns	42.4 *	100
Increased Irritability	35.9 *	100
Headaches	41.4 *	100
Dizziness	62.4	100
Rigor	51.8	100
Abdominal Bloating	51.3	100
Nausea	53.8	100
Loss of Appetite	60.1	100
Bowel Pattern Changes	46.2 *	100

NOTE:

* 50% or more of the patient sample that endorsed this symptom also attributed it to a UTI at least some of the time.

Values in the table that are *less* than 50% represent a minority of the endorsers *never* attributed the symptom to a UTI.

Table 5 shows that the controls attributed just one of the USQNB-IC items to UTI in any appreciable numbers: “burning on passing urine”. “Blood in urine”, “cloudy urine”, and “abdominal pain” were the items with the lowest proportion of controls attributing the symptom to a UTI. By contrast, the *majority* of the patient groups attributed 16 of these 29 symptoms to UTIs–these are indicated with an asterisk (*). Because of how participants in both groups were recruited, it is supportive of face and content validity claims that most patients with experience with UTIs do attribute most items to having a UTI while participants who were recruited specifically because they have limited experience with UTIs do not attribute these symptoms to having a UTI. Also, members of our focus groups for developing the instrument originally were from the patient group, not from the divergent validity “control” groups. Thus the higher likelihood of attributing these symptoms to having a UTI in our target patient group also supports the reliability and validity of these items.

Taken together, these results suggest that our instrument possesses important validity evidence according to the COSMIN criteria. Specifically, the results present several lines and sources of evidence of face, content, criterion, convergent, and divergent validity for these items; reliability evidence also arises from the results considered all together.

## Discussion

The purpose of this study was to describe the measurement properties of a new patient-*centered* patient reported outcome instrument for urinary signs and symptoms in people with neurogenic bladder and who use intermittent catheterization, the USQNB-IC. Overall, our validity evidence addresses the following COSMIN criteria:

1. Face, content, convergence validity, reliability: All items—which were generated by patients themselves and then integrated with clinician input (see Fig 1)–were recognized and endorsed by at least 10% (most were endorsed by at least 50%) of our target patient populations: those with neuropathic bladder who use intermittent catheterization, and who experience frequent UTIs (Table 3).
2. Divergent validity, reliability: Few items were endorsed by even 50% of any of the control groups (Table 4), suggesting that the items are more descriptive for our target patient group and less descriptive of the experiences of those outside that group. This divergent validity evidence also supports claims of reliability, namely, that the items describe what they are intended to describe.
3. Convergent, divergent validity: The total number of items endorsed by patient groups were similar to each other, but were more than double, on average, the total number endorsed by control groups (Table 5).
4. Criterion validity: Of those endorsing any item, one item (burning on passing urine) was attributed to a UTI by 50% of the controls but every other item was *not* attributed to a UTI by 75% or more controls (most items were not attributed to a UTI by 100% of controls). Burning on passing urine is a classic symptom of a UTI, offering additional convergent validity evidence, as well as criterion validity evidence, from the control groups.

This instrument is substantially (and substantively) different from any other: it was developed using explicitly patient-centric methods, is specific to the lived experience of the neurogenic bladder population, and the focus of these items is on infection-related symptoms. Urinary symptoms tools and health quality of life scales generally fall under the domains of female/general urinary incontinence, male urinary symptoms, and specific urinary symptoms (nocturia, overactive bladder, urgency, patient perception of bladder condition, and intermittent catheterization).[15] A recent (2016) systematic review of patient reported outcome measures for neurogenic bladder and bowel revealed heterogeneity in the current patient reported outcome measures in this area and a clear focus on quality of life. While the Medical Outcomes Study SF-36 was the most frequently utilized tool, only three bladder or bowel specific measures were identified: the Qualiveen, FICQoL, and the QoL-BM—all quality of life, and not symptom, measures.[18] Another systematic review examined and compared validated questionnaires for people with neurogenic bladder due to SCI and multiple sclerosis. Of 18 questionnaires identified, 14 were for people with MS, 3 for people with SCI, and 1 was general.[17]

The only peer reviewed, published symptom scale that we identified for people with neurogenic bladder is the NBSS (Neurogenic Bladder Symptom Scale).[15,27] In the development of this generic scale, 266 items were generated from a search of the existing/published (not specific to neurogenic bladder) measurement tools. Of these, only two were specific to urinary tract infection. A multi-disciplinary team reduced the original set of 266 items, and interviews were conducted with consumers with SCI and MS, who identified urinary incontinence, UTI, urgency, and bladder spasms to be dominant issues for them.[15] While our instrument is also specific to individuals with NB, our measure differs significantly from the NBSS in terms of the origins of our items (i.e., see Tractenberg et al.[16] and Fig 1) and their scope. As noted, the development of our measure was fundamentally different from that of the NBSS, as our core information/symptoms is based in patient report, as opposed to deriving from clinician- or evidence-defined reports.[16]

The USQNB-IC has the potential to meaningfully advance the quality of health care in the population of people with neurogenic bladder, a population in which UTIs are common, and likely over-treated with antimicrobials.[28] This over treatment may be due, in part, to our lack of understanding of the different clinical components contributing to UTI diagnosis (symptoms, inflammatory markers, bacterial load). There is currently broad consensus that only symptomatic UTI should be treated,[28–30] however the term “symptomatic UTI” is not well defined. We expect that, deriving from the patients’ experience, the USQNB-IC includes symptoms that may be attributable to infection, as well as those that precede the infection, possibly signaling an increase in susceptibility or vulnerability. Most scientists and clinicians agree that fever is a symptom[28,30,31] of an infection, and when no other source of infection can be identified, UTI is a common source. However, difficulties remain in differentiating other symptoms more likely to be indicative of UTI versus non-infectious or non-urinary symptoms. According to the International SCI UTI Basic Data Set[32], signs/symptoms of UTI include: fever, incontinence (onset or increase in episodes, including leaking around catheter), increased spasticity, malaise (lethargy or sense of unease), cloudy urine (with or without mucus or sediment) with increased odor, pyuria, discomfort or pain over the kidney or bladder or during micturition, autonomic dysreflexia, and ‘other’.[32] The International SCI UTI Data Set is intended to be a standardized collection tool for the minimal amount of information related to a possible UTI. As such, these symptoms are derived from clinical consensus and are appropriately broad for the purpose of a standardized data set. These nine symptoms (which are a subset of the ones our focus groups and clinicians identified, except for autonomic dysreflexia), are not intended to be specific and comprehensive enough to be used in routine clinical practice. By contrast, the USQNB-IC has 29 urinary symptoms that encompass the authentic lived experience of urinary symptoms by the patient with neurogenic bladder—as well as these data set items—so these might be useful for routine clinical practice as well as research and potentially, self-management by patients.

There is significant overlap between the International SCI UTI Data Set-identified signs/symptoms and the USQNB-IC: “fever”, “incontinence”, “malaise”, “increased spasticity”, “discomfort”, and “cloudy urine” are present in both the International SCI UTI Data Set and our patient-centric list; however “incontinence” is further elaborated on by patients with additional descriptive symptoms such as “increased frequency of spasms”, “increased urgency”, “increased catheterization”, “increased incontinence”, and “increased urine leakage.” Similarly, in addition to “malaise”, the USQNB-IC captures constitutional symptoms identified by our focus groups as distinct, such as “fatigue”, “generally not feeling well”, “muscle aches”, “altered sleep patterns”, “increased irritability”, “dizziness”, and “loss of appetite”. We are in the process of deepening our understanding of how all of these patient- and clinician- focus group-identified symptoms on the USQNB-IC can be used by patients and clinicians, as well as investigators studying potential intervention and self-management strategies.

Although we have multiple lines of evidence supporting claims that the instrument is valid, the study has some limitations and considerations. Foremost among these is that we did not include a control group that did not have neurogenic bladder but did have as many urinary problems as our neurogenic bladder patient groups did. However, a proxy for this evidence is the consensus-based guidelines for diagnosing urinary tract infection set out by the Infectious Diseases Society of America (IDSA).[26] These guidelines are derived from clinician experience with general UTI patients; those with neither mobility impairment nor neurogenic bladder and *with* UTIs. Testing the convergent and divergent validity of these items with an equally symptomatic group that has no mobility impairment is an important next step in our validation efforts, although our earlier work[16] shows that consensus-based guidelines, which are not based on individuals with neurogenic bladder and so do derive from an equally symptomatic group that has no mobility impairment, are not as frequently endorsed among persons with neurogenic bladder as those derived from our focus groups. Five of these IDSA guideline symptoms are among the lowest endorsed items of the USQNB-IC in the patient population.

A second limitation is that we asked whether each item had been experienced within one year, although the intended use of the instrument is for a much tighter time window, such as one or two weeks. Because this was a preliminary validation study, we wanted to ensure that we captured patient experiences with these symptoms; we asked those in the national sample to estimate the frequencies of each item within the past year to get an idea of whether using this instrument with shorter time frames would ever be feasible. We also asked about the past year to capture evidence of any possible seasonality or other longer-term fluctuations in symptoms—as well as to ensure that we did not obtain misleading evidence about the relevance of these symptoms by artificially limiting the recall window. Other research questions our team is investigating, for which this instrument was specifically created, do involve administration of the USQNB-IC every week, so eventually we will have a sense of endorsement rates over shorter time frames once that study is completed. For these preliminary results, to increase the likelihood of capturing interpretable data on these signs and symptoms, the one-year timeframe was essential, even if these endorsement rates over that period preclude us from determining, for example, which of these items occur (or are most likely to occur) together.

A further consideration relates to our exclusion and inclusion criteria: while our focus groups were solely comprised of community-dwelling individuals, the national survey did not exclude respondents with any of the following conditions from participation: 1) known genitourinary pathology beyond neurogenic bladder (i.e., vesicoureteral reflux, bladder or kidney stones, etc.); 2) use of prophylactic antibiotics; 3) instillation of intravesicular agents to reduce UTI (i.e., gentamycin); 4) psychologic or psychiatric conditions influencing the ability to follow instructions; and 5) participation in another study in which results would be confounded. Although these were all exclusion criteria for the focus groups, it is possible that our national sample included respondents with some of these potentially confounding factors. While this may enhance the generalizability of our national results, the relationships between these patient characteristics and responses on the USQNB-IC are unknown at this point.

Finally, these data suggest that having neurogenic bladder does not by itself confer the UTI/urinary problems that all our SCI, SB, and CG reported, because we found a control sample of individuals who also have neurogenic bladder, but who endorsed the same signs and symptoms much less often than those who do have frequent UTIs. Thus, we can now incorporate this fact in the designs of future studies and interventions, knowing that an individual has neurogenic bladder alone is too broad to identify a target patient population; further, tools like the NBSS may not be sufficiently specific for work in this population. Our own work continues with efforts to develop new instruments (following the same methods outlined here) for patients with neurogenic bladder who manage their bladders with either indwelling catheters or by voiding.

## Conclusions

This instrument was developed as a specifically *patient-centered* patient reported outcome, and so is consistent with principles of “valuing the patient perspective”, and maintaining a “culture of patient centeredness in research”.[4] However, this patient-centered approach is inconsistent with formal methods for instrument development (i.e., those laid out by Bollen, 1989)[21] that lead to the very measurement properties that are of greatest interest (as per Mokkink et al. 2010).[20] Our work in this area (urinary symptom detection and management for neurogenic bladder) has balanced these two perspectives, and our preliminary validity evidence suggests that both can be integrated to yield a valid PC-PRO instrument. Our next objective is to explore the best scoring approaches to this instrument, keeping the balance in mind, but also considering that we intend this instrument for use by patients in their own self-management, and by clinicians in their management of (collaboration with) patients who are also using the instrument.

## Supporting information

S1 Data Data for these analyses. (CSV)
